# Community Versus Facility-Based Services to Improve the Screening of Active Hepatitis C Virus Infection in Cambodia: The ANRS 12384 CAM-C Cluster Randomized Controlled Trial—Protocol for a Mixed Methods Study

**DOI:** 10.2196/63376

**Published:** 2024-11-20

**Authors:** Emilie Mosnier, Olivier Ségéral, Sansothy Neth, Luis Sagaon-Teyssier, Dyna Khuon, Chan Leakhena Phoeung, Sovatha Mam, Chhingsrean Chhay, Kimeang Heang, Jean Charles Duclos-Vallée, Vonthanak Saphonn

**Affiliations:** 1 Aix Marseille Université, Institut national de la santé et de la recherche médicale (INSERM), Institut de recherche pour le développementIRD, Sciences Economiques & Sociales de la Santé & Traitement de l’Information Médicale (SESSTIM) Aix Marseille Univ, Aix Marseille Institute of Public Health (ISSPAM) Marseille France; 2 University of Health and Sciences Phnom Penh Cambodia; 3 HIV Unit Infectious Diseases Department Geneva University Hospitals Geneva Switzerland; 4 Hepato-Biliary Department Paul Brousse Hospital, Assistance Publique - Hôpitaux de Paris (APHP); Institut national de la santé et de la recherche médicale (INSERM) U1193 Université Paris-Saclay; University Hospital Federation (FHU) Hepatinov Villejuif France

**Keywords:** hepatitis C virus, HCV, community-based intervention, cluster randomized controlled trial, Cambodia, cost-effectiveness analysis, qualitative analysis

## Abstract

**Background:**

In Cambodia, hepatitis C constitutes a significant public health challenge, particularly among older adults (>45 years) for whom prevalence is estimated to be 5%. To facilitate the elimination of hepatitis C among the general population, enhancing access to screening and treatment is imperative. In this regard, the evaluation of community-based screening programs emerges as a crucial step toward improving health care accessibility.

**Objective:**

This study aims to assess the comparative efficacy of a community-based versus a facility-based approach in enhancing the uptake of hepatitis C antibody testing among the general population older than 40 years of age in Cambodia.

**Methods:**

The CAM-C (Community Versus Facility-Based Services to Improve the Screening of Active Hepatitis C Virus Infection in Cambodia) study uses a cluster-randomized controlled trial design across two Cambodian provinces to compare community-based and facility-based hepatitis testing interventions. Sampling involves a multistage cluster approach, targeting individuals older than 40 years of age due to their higher prevalence and risk of chronic hepatitis complications. This study incorporates a qualitative analysis of acceptability and a cost-effectiveness comparison. Interventions include facility-based testing with subsequent referral and community-based testing with direct in-home assessments. Follow-up for positive cases involves comprehensive management and potential direct-acting antiviral treatment. This study aims to identify a significant increase in testing uptake, requiring the screening of 6000 individuals older than 40 years of age, facilitated by a structured sampling and intervention approach to minimize contamination risks.

**Results:**

The final protocol including the quantitative, qualitative, and cost-effectiveness part of the study was registered and was approved in 2019 by the National Ethical Cambodian for Health Research. Inclusions were completed by mid-2024, with analyses starting in May 2024.

**Conclusions:**

Using a mixed methods approach that combines a robust methodology (cluster-randomized controlled trial) with a cost-effectiveness analysis and qualitative research, such a study should provide invaluable information to guide the Ministry of Health in its hepatitis C virus screening strategy and move toward elimination.

**Trial Registration:**

ClinicalTrials.gov NCT03992313; https://clinicaltrials.gov/study/NCT03992313

**International Registered Report Identifier (IRRID):**

DERR1-10.2196/63376

## Introduction

Hepatitis C virus (HCV) infection is a major public health concern, affecting millions of individuals worldwide. In recent years, several countries have launched ambitious national programs aiming at eliminating HCV as a public health threat [1,2]. These programs use a range of strategies including widespread screening, access to affordable treatment, harm reduction measures, and public awareness campaigns. Current efforts to eliminate hepatitis C at the national level vary in scope and implementation strategies. Countries, like Egypt, Australia, Georgia, and Iceland, have emerged as leaders in the global fight against HCV, demonstrating significant reductions in disease prevalence and incidence [3,4]. These countries have adopted multifaceted approaches that encompass mass screening, treatment access, harm reduction interventions, and public engagement [4]. Successful outcomes have been achieved through proactive government policies, collaboration with health care providers and community organizations, and innovative financing mechanisms [1,5].

One of the significant recent advancements is the availability of direct-acting antivirals (DAA), with cure rates approaching 90% to 95% along with their good tolerance. These treatments are expected to change the dynamic of HCV infection worldwide and give hope for eradication. However, the impact of DAA in the general population is completely related to the population-level cascade of care: screening programs must reach undiagnosed individuals, diagnosed individuals must be linked with care, and people engaged with care must be assessed, receive treatment, and be cured. In 2016, in the Western Pacific region, according to the World Health Organization (WHO), only 21% of people living with HCV have been diagnosed and 2% started treatment. There is a need to address how different interventions apply to the context of well-tolerated, simple, oral treatment regimens. The Georgia HCV elimination program has recently reported promising results [6] highlighting that “high-quality screening, innovative linkage-to-care strategies, and cost-effective and simplified diagnostic and treatment regimens are needed” to reach the elimination target.

In Cambodia, the burden of viral hepatitis exceeds the combined burden of HIV and tuberculosis. Estimates suggest that 3% of the population (approximately 475,000 individuals) live with chronic hepatitis B virus (HBV), and 1.6% (around 257,000 individuals) with chronic HCV [7]. These infections are the predominant causes of liver cirrhosis and liver cancer–related fatalities in the country, leading to roughly 2300 deaths annually. Liver cancer, primarily attributed to viral hepatitis, has become the foremost cause of cancer death in Cambodia [8]. A 2018 seroprevalence survey by Médecins Sans Frontières (MSF) nongovernmental organization and Epicentre highlighted a critical awareness gap; 64% of respondents and 57% of seropositive participants had never heard of HCV. Moreover, the prevalence among older adults (>45 years) was significantly higher (5.1%) compared to those younger than 45 years old (0.6%) [9].

A recent study indicated that people living with HIV are at a heightened risk of contracting HCV, with a prevalence rate of 6.8%. This study also identified two significant risk factors for HCV infection: having a family member positive for HCV and the use of intravenous medication in the past 5 years [10]. These efforts underscore the complexity of the hepatitis epidemic in Cambodia and the multifaceted approach required to address it. The challenges in eliminating hepatitis in Cambodia include not only the provision of medical treatments but also the need for increased public awareness, improved diagnostics, and the integration of hepatitis screening and care into the broader public health infrastructure including communities’ health care workers.

While there has been considerable progress in access and affordability to diagnostic and treatments for lower-middle-income countries there is a sheer lack of international or domestic financing for a robust program for patients chronically infected with hepatitis B or C [10,11]. In 2018, a modeling study led by Bristol University using data from the MSF pilot project demonstrated the cost-effectiveness of a public health intervention [12]. This analysis was made at the time of higher prices of HCV commodities and estimated the cost for the elimination of about US $60 million by 2030 [13]. The 12-week DAA treatment for HCV chronic patients would not only prolong thousands of lives but also offer a direct cost saving of US $18.3 million. Discussions with UNDPA (United Nations Department of Political Affairs) are underway to help Cambodia benefit from lower prices. In the past 2 years, the Cambodian government has committed to the national elimination program by 2023, allocating US $1 million per year for screening tests and treatment.

We hypothesize that enhancing hepatitis care necessitates identifying and tailoring effective screening strategies for the general population, particularly prioritizing those older than 40 years of age due to their vulnerability to severe hepatitis outcomes. Our approach advocates for integrating serological and virological assessments to boost the precision of active infection detection. Additionally, we advocate for a community-based testing model, leveraging rapid diagnostic tests (RDT) and dried blood spot (DBS) synergies, to potentially surpass traditional facility-based methods in accessibility and patient engagement. The main objective of this study is to compare the effectiveness of a community-based intervention to a facility-based intervention to improve the antibody-testing uptake of HCV infection among the general population older than 40 years of age in Cambodia.

## Methods

### Study Design

The CAM-C study is a 2-arm cluster randomized controlled trial, in which clusters are defined as a group of 50 households corresponding to the service population of the community health care workers (CHWs). This study includes a qualitative investigation of the acceptability of both strategies, as well as a cost-effectiveness analysis comparing the community-based and facility-based arms with each other and with the status quo.

### Study Overview and Sampling

This protocol outlines a cluster randomized controlled trial designed to evaluate the effectiveness of facility-based testing interventions (Arm 1) compared with community-based interventions (Arm 2) in improving the detection of people with HCV active infection.

**Figure 1:**
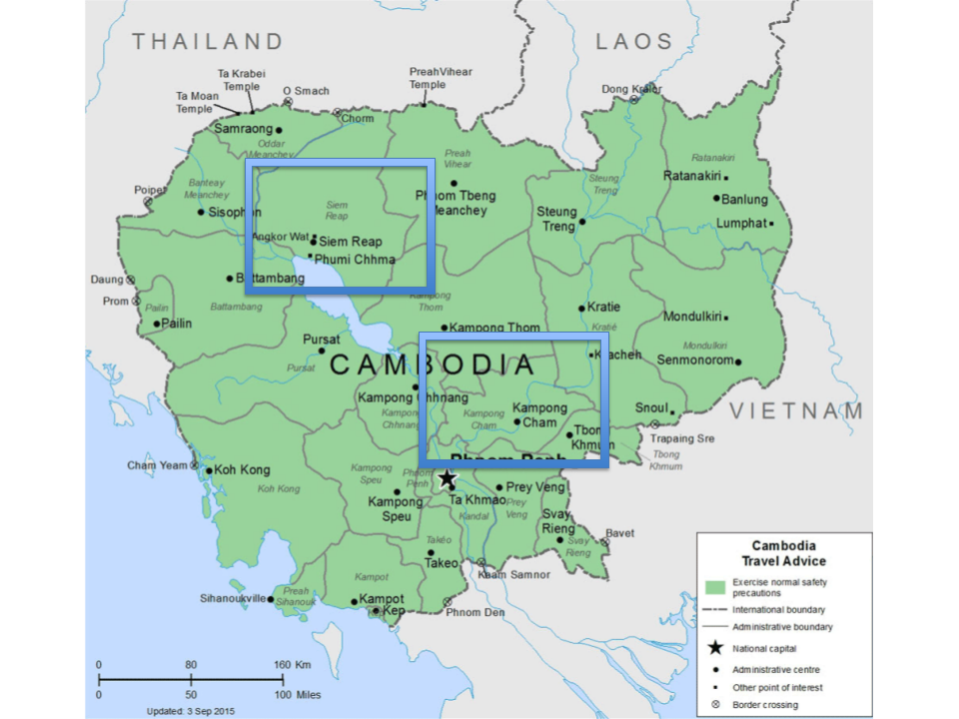
Study area Siem Reap and Kampong Cham provinces.

The research was conducted across two separate provinces in Cambodia: Siem Reap and Kampong Cham. Both provinces have a population of approximately 1 million, each served by a provincial hospital. Siem Reap is organized into 4 operational districts (ODs), 103 communes, 5 referral hospitals, and 88 health centers. Kampong Cham, on the other hand, comprises 9 ODs, 107 communes, 9 referral hospitals, and 98 health centers (Figure 1).

For the purpose of this study, one OD in Siem Reap and two ODs in Kampong Cham have been selected. These districts have a comparable number of villages and inhabitants and are equidistant from their respective provincial health departments. Each district is further segmented into communes, which are then divided into villages, and subsequently into groups, typically consisting of 50 households each. One such group will be designated as a cluster.

To further reduce contamination risks between arms in terms of intervention-related activities, four geographically distinct areas, separated by nonparticipating communes, have been chosen in each province. These areas were randomly assigned to either Arm 1 or Arm 2. A multistage cluster sampling approach was used for participant selection within each geographic area. Initially, a probability proportional to size sampling method was used to select 10 villages in each geographic area. A preliminary mission allowed us to verify the locations of households within these villages, during which demographic data, including inhabitants’ ages, were collected. Subsequently, a simple random sampling method was applied to select 100 households per village from the 10 chosen villages. Finally, all individuals older than 40 years of age within these households were invited to participate in the study. This structured approach ensures a comprehensive assessment of the proposed testing interventions within the target populations. The inclusion and exclusion criteria are provided in Textbox 1.

**Inclusion and exclusion criteria.**

**Inclusion criteria**

- All persons older than 40 years of age
- Residing in the study area
- Informed consent obtained with oral information given and explained and the consent form signed by the participant

**Exclusion criteria**

- Known hepatitis C virus (HCV) status with previous HCV treatment
- Severe disease present at inclusion involving life-threatening
- Concurrent participation in any other clinical study without written agreement of the two study teams

For the qualitative part of the study, key participants for the interviews included leaders in the health sector, such as heads of health centers and ODs, alongside health care providers directly involved in the intervention, including health centers staff, community health workers, and village health support groups.

### Sample Size

The objective was to discern a significant increase in testing uptake from 60% in facility-based settings to 80% in community-based settings among individuals older than 40 years. The calculation was based on data from the 2014 Cambodia Demographic and Health Survey report, which indicates an average rural household size of 4, with 25% of individuals being older than 40 years, resulting in an estimated cluster size of 50 [14].

Key assumptions for this calculation included an intraclass correlation of 0.01, a significance level of 5% (α=.05), and a study power of 80% (1-β). The design effect was set at 1.5, reflecting the expected variance inflation due to cluster sampling.

To achieve the desired power for detecting the anticipated difference in testing uptake between the two study arms, the study required screening at least 300 patients for HCV RNA. This implied screening at least 6000 individuals older than 40 years by RDT on the basis of an estimated 5% positivity rate for HCV antibodies. Given an expected median testing uptake of 60%-70%, the study plans to propose testing to 8000 participants, distributed across 160 clusters (80 clusters per intervention arm), to ensure reaching the target of 6000 screened participants.

### Intervention

**Figure 2:**
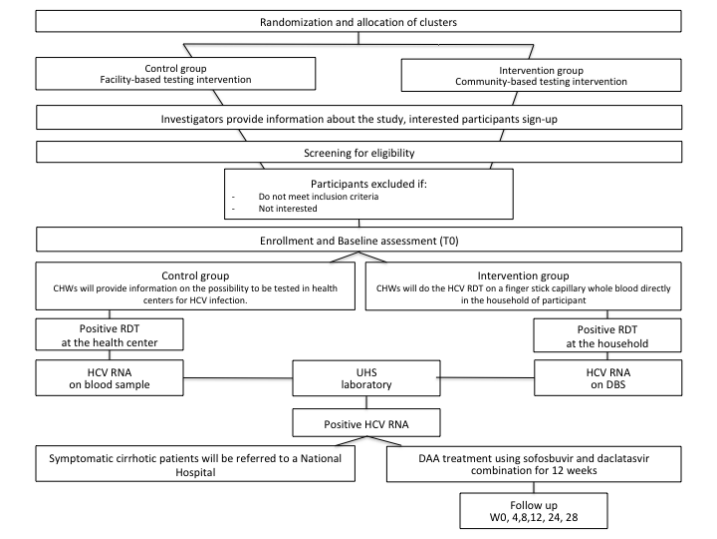
Study design of the intervention. CHW: community health care worker; DAA: direct-acting antiviral; DBS: dry blood spot; HCV: hepatitis C virus; RDT: rapid diagnostic test; UHS: University of Health and Science, Phnom Penh.

The study design of both interventions is resumed in Figure 2.

#### Arm 1: Control Group, Facility-Based Testing Intervention

In this arm, CHWs disseminated face-to-face information in selected households about HCV testing opportunities at health centers through information sheets and dedicated leaflets. Interested participants received a voucher to attend a referral health center for HCV testing. Upon consenting formally at the health center, individuals underwent HCV testing via RDT using finger stick capillary whole blood, with results available within 15 minutes. Positive RDT participants had an immediate collection of a 5-mL whole blood sample (using an ethylenediaminetetraacetic acid tube) at the health center, which was forwarded to the provincial hospital laboratory for plasma extraction. The plasma was sent to the Pasteur Institute of Cambodia for HCV RNA analysis, with the outcomes relayed back to the health center. Health center nurses communicated the results to the participants and facilitated referrals for those with active infections, ensuring follow-up through systematic appointments and phone calls for nonattendees.

#### Arm 2: Intervention Group, Community-Based Testing Intervention

In this arm, CHWs, after receiving specialized training, performed HCV RDTs directly in participants’ homes using finger stick capillary whole blood. Information was provided through the same information sheets and leaflets as the facility arm, and consent was obtained prior to testing in participants’ homes. Should household testing face structural or societal hurdles, alternate testing locations within the village may be used. Coordination with village heads ensured population awareness, with a provision for a second visit for absentees during the initial visit; no further attempts were planned after a missed second visit. For participants testing RDT positive, 5 blood spots were collected immediately on DBS cards, securely packaged with the participant’s ID number, and dispatched to the Pasteur Institute of Cambodia for HCV RNA extraction and amplification. Results were communicated back to each village’s referral health center, where nurses were responsible for result disclosure to participants and subsequent referral for those with active infections, supplemented by systematic appointments and phone calls for those who did not attend.

### Follow-Up

Participants with negative HCV RNA results received specific counseling tailored to their situation. Participants with positive HCV RNA were scheduled for a consultation at the provincial hospital. If a consultation was not attended within 4 weeks, an SMS text message reminder was dispatched to the participant. Trained general practitioners, with potential phone support from hepatologists, conducted the consultations. The initial assessment for these participants encompassed questionnaires covering risk behaviors and socioeconomic status, a clinical examination, blood sampling, and a liver ultrasound.

Patients displaying symptoms indicative of cirrhosis, such as ascites, collateral circulation on the abdominal wall around the umbilicus, encephalopathy, jaundice, or those with hepatocellular carcinoma, were referred to the hepatology unit in a national hospital in Phnom Penh (Calmette hospital).

For other patients, DAA treatment was offered, pending a review of creatinine levels and potential drug-drug interactions. The study used nonexperimental drugs, specifically a daily regimen of sofosbuvir (400 mg) and daclatasvir (60 mg) for 12 weeks. WHO-prequalified generic drugs from Hetero Labs Limited, comprising a daily dose of Sofosbuvir (400 mg) and Daclatasvir (60 mg) were used. DAA treatment was initiated at the first provincial hospital consultation, with monthly follow-ups thereafter. All adverse events were assessed by the investigator and documented regardless of the possible causality with the concomitant treatments. Adherence was evaluated every month using a questionnaire and visual analog scale. Biological follow-up during the treatment is reported in Multimedia Appendix 1.

### Blood Sample Collection and Tests

Upon confirmation of HCV RNA positivity, further tests were conducted at the initiation of DAA therapy, including screenings for HBsAg, HIV syphilis duo, and a range of blood and biochemical analyses. The rapid tests used in the study were Alere Determine, HBsAg, Bioline HCV test, and Bioline HIV/Syphilis Duo test.

Subsequent tests are scheduled at weeks 4, 8, 12, and 24, with varying focuses, such as complete blood counts, serum transaminases, and serum creatinine levels, culminating in a plasma HCV-RNA viral load test at week 24.

The study differentiates between Arm 1 and Arm 2 in terms of HCV RNA testing methods, with Arm 1 using plasma and Arm 2 using DBS for sample collection and transport to the Pasteur Institute laboratory for analysis. Post screening, all samples are slated for destruction, except for those designated for the biobank.

For the biobank, samples collected during therapeutic follow-up at weeks 0, 12, and 24 were stored at the University of Health and Science’s Rodolphe Merieux laboratory. Plasma from these samples was aliquoted and frozen, with a dedicated database for sample registration. These samples are preserved for 15 years post participant consent, facilitating future research and analysis.

### Data Collection

Clinical research assistants who obtained signed informed consent from participants in their native language collected data for this study on the field. For qualitative data, interviews were recorded, transcribed, and translated from Khmer into English for analysis. The data collection encompassed various aspects, including the cost-effectiveness analysis of the two study arms (community-based and facility-based interventions). This included detailed cost data such as the time spent by human resources, salaries, the costs of tests, and the treatments in each study arm. All collected data were then anonymized to ensure participant confidentiality and privacy.

### Statistical Plan

The demographic and socioeconomic characteristics of participants at inclusion will be detailed for the total sample and comparatively between arms by using chi-square, Student *t* test, or Wilcoxon tests as applicable (eg, comparing proportions, means, or medians). The comparison between arms in terms of demographic, socioeconomic characteristics, and the distribution across provinces will provide information about potential significant differences between arms. In this case, statistical methods, such as inverse probability weighting, will be implemented in order to ensure the comparability of arms, that is, avoiding sampling or selection biases introduced by implementation issues, participants’ behaviors, etc [15,16]. The primary outcome will focus on the percentage of participants tested for HCV RDT and informed of their status within the total eligible population in the intervention regions, with comparisons drawn between the two arms. Secondary outcomes will assess the percentage of persons tested for both HCV RDT and HCV RNA, the number of individuals with active HCV infection who received and understood their results, and the number of persons with at least one consultation at the provincial hospital, all compared between the study arms. The ANOVA will be used to identify significant differences in primary and secondary outcomes between the clustered trial arms, supplemented by confidence levels, intervals, and effect sizes to elucidate the data. An Analysis of Covariance model will analyze the main outcome by considering the baseline as a covariate and will test for associations between covariates to provide a thorough understanding of the intervention’s impact. A logistic mixed model will be used to assess the associated factors (odds ratio and 95% CI) for the primary outcome. This will allow accounting for cluster intraclass correlation and disentangle the variability of the outcome due to geographical (eg, provincial) and individual differences [16,17].

### Cost-Effectiveness Analysis

The cost-effectiveness analysis of this study compares the value of mobile HCV testing services versus incentivizing health center testing, focusing on individual and societal benefits. It tackles methodological challenges by carefully evaluating costs associated with DAA, the analysis perspective (ie, health system and individual), time horizon, and HCV-related diseases including nonmedical expenses. Costs are broken down by intervention arm, covering community information, HCV RDTs, blood sample collection, and transportation, with additional costs for CHWs mobilization in community-based screening. Common costs across both arms include further biological tests, DAA treatment, concomitant drugs, outpatient consultations, and inpatient care. Special attention is given to cirrhotic patients and nonresponders, accounting for specific tests and treatments. The main effectiveness criterion corresponds to the primary outcome of the study (ie, the proportion of participants with HCV RDT tests and informed of their status among the total eligible population). This analysis aims to provide a detailed understanding of the economic implications of each testing strategy.

### Qualitative Study

The qualitative component of the study aims to explore the acceptability of both facility-based and community-based HCV testing strategies, engaging approximately 94 participants. The methodology includes semistructured interviews with about 30 health sector leaders and health care providers, including heads of health centers, community health workers, and village health support groups, to understand their perspectives on HCV testing’s context, strengths, and challenges. Additionally, 8 focus group discussions will be conducted, involving around 64 participants from the community, divided equally between those who accepted and those who declined HCV testing. These discussions aim to gather diverse viewpoints on testing strategies, barriers to testing, and suggestions for increasing testing uptake in Cambodia. The study’s structured approach, using a mix of interviews and focus groups, is designed to offer comprehensive insights into the feasibility and acceptability of HCV testing interventions, thereby informing effective HCV management and control strategies.

### Ethical Considerations

In this study, patient participation was strictly voluntary, with comprehensive information provided about the study’s aims, procedures, duration, risks, benefits, and possible discomforts. This information was conveyed both verbally and through a written sheet in Khmer language. Consent was given before any study-related exams, with participants signing a consent form, a process overseen by the investigator. Consent forms were securely stored, and participants received a copy. The process varies slightly between the 2 study arms, with consent collected at health centers for Arm 1 and in participants’ households for Arm 2. A separate consent process was also in place for the therapeutic phase at the provincial hospital and the qualitative part of the study. The protocol received approval from the National Ethical Cambodian for Health Research on February 25, 2019 (approval 047). This protocol has also been registered with ClinicalTrials.gov (NCT03992313).

## Results

This project has received funding from the ANRS Emerging Infectious Diseases (ANRS MIE) award ANRS 12384. The final protocol including quantitative, qualitative, and cost-effectiveness parts of the study was registered and approved in 2023 by the National Ethical Cambodian for Health Research. Participant enrollment was completed by mid-2024, and analyses commenced in May 2024. We will publicize our findings through peer-reviewed studies, conference presentations, and recommendations to the local and national public health institutions.

## Discussion

### Expected Benefits of the Study

The Royal Government of Cambodia, in alignment with the WHO’s global objectives, has committed to the ambitious goal of eliminating HCV and HBV by 2030. Since the 1990s, the Cambodian government has expanded access to systematic screening of blood donations for HBV and HCV, as well as the widespread adoption of single-use syringes. The latter measure is especially critical, given that HCV transmission is predominantly associated with parenteral interventions, surgeries, or endoscopies prior to the year 2000 [18]. Populations at greatest risk appear to be individuals older than 40 years of age [19]. In June 2018, the Ministry of Health established a Viral Hepatitis Technical Working Group, led by the Director of the Communicable Disease Control Department. This group was tasked with developing a Viral Hepatitis National Strategic Plan and guidelines policies based on scientific evidence and enhancing the accessibility of diagnostics and treatments within the public health system. Hepatitis was listed as a priority area for action within Cambodia’s most recent national strategic health plan (2016-2020), and in 2017, the National Center for HIV/AIDS, Dermatology and STDs secured funding from the Global Fund (using underspend on their grant) to diagnose and cure HIV/hepatitis C coinfected patients enrolled in their antiretroviral therapy program.

One of the main issues identified within the National Technical Working Group was the difficulty in reaching at-risk people for HCV screening. In 2020, using a health center-based approach, the MSF project reached a screening coverage of 40% of the adult population older than 45 years of age estimated in need of HCV treatment in 3 ODs of the province of Battambang [20]. This excellent result in the context of the COVID-19 pandemic is, however, insufficient to reach the 80% coverage target required for elimination. A community-based approach, offering screening directly in the homes of those concerned, could therefore be an effective strategy for increasing screening coverage.

Within this ambitious framework, this project aims to provide innovative data on the effectiveness of a community-based screening method, as well as the characteristics and factors associated with screening effectiveness in households or health centers. Additionally, qualitative data will complementarily highlight the barriers and facilitators to scaling up and implementing such interventions through a multi-level evaluation that includes participants, those who opt out of testing, community workers, and health care providers. Finally, the cost-effectiveness study will offer updated and robust data to support advocacy and cost estimation for scaling up, contributing to the national eradication strategy.

### Limitations and Strengths

The mixed method study protocol on community versus facility-based services for HCV screening in Cambodia presents a robust design, yet it encompasses inherent limitations and strengths.

One limitation is the potential for selection bias, as the study relies on voluntary participation, which might not fully represent the broader population’s perspectives on HCV testing. Additionally, the qualitative component’s reliance on self-reported data may introduce response bias, where participants might provide socially desirable answers. The study’s cluster-randomized design could also face operational challenges, such as logistical issues in remote areas, which might affect the uniformity of intervention implementation.

On the other hand, the study’s strengths are significant. The mixed methods approach allows for a comprehensive understanding of the acceptability and feasibility of HCV testing strategies, combining quantitative data’s robustness with qualitative insights’ depth. The inclusion of a diverse participant pool from different health sectors and the community enhances the generalizability of the findings. Moreover, incorporating a cost-effectiveness analysis will allow for better scalability and sustainability of the interventions based on the results, directly addressing the practical challenges of HCV screening in Cambodia and providing valuable data to inform national health strategies. This approach, grounded in local realities, positions the study to offer actionable recommendations for enhancing HCV care and control in Cambodia and similar settings.

### Comparison With Prior Work

The question of “who to test” and “how to test” in low and middle-income countries was discussed in a recent study [21]. WHO recommended that “HCV serology testing be offered to individuals who are part of a population with high HCV seroprevalence or who have a history of HCV risk exposure or behavior” [22]. This recommendation includes persons who have received medical or dental interventions in health care settings where infection control practices are substandard which was the case in Cambodia before the 2000’s. Targeted HCV testing interventions to individuals who are a part of risk groups for HCV infection or who have a history of HCV risk behaviors seems to be effective in diagnosing cases and increasing treatment uptake [23] but the reported programs are mainly heterogenic, and the majority did not use a comparison group [24]. However, many countries in Europe and the United States recommend a systematic population-based screening for groups at risk of infection. In the United States, excepting classic at-risk groups (injecting drug use, hemodialysis, and prisoners), HCV infection is most prevalent among individuals born between 1945 and 1965 (baby-boomer population) [25]. Since 2012, Communicable Disease Control has recommended HCV screening in this population. In France, recommendations changed in 2014: HCV screening must be done for all men aged 18-60 years, with also a particular warning for the baby-boomer population, as many persons are still undiagnosed in 2014 in this population [26]. Screening could be done in different medical structures [27-30] and could easily be integrated into routine primary care [31].

Nevertheless, all these studies were conducted in high-income countries, and data in low-income countries are scarce. Rapid diagnosis tests are available in the majority of these countries but targeted testing interventions are related to the country’s epidemic situation and the health care structures. Community approach with testing at a household level was reported effective in Egypt to ensure both high uptake of testing, and equity in access regardless of sex, age, income level, or stage of disease [32]. As recommended by WHO, operational research is needed to evaluate different approaches to increase the reach and uptake of simplified screening services close to the population [33]. Indeed, a comprehensive and integrated approach to the elimination of HCV at a community level must incorporate both preventive approaches to reduce transmission and new infections, and testing and treatment to reduce both the burden of disease and associated morbidity. A prompt linkage to care to ensure access to treatment is essential.

### Conclusions

The CAM-C study aims to gather data on the effectiveness of a community-based screening strategy for hepatitis C in a high-prevalence general population country. These findings will be used to tailor public health interventions and to inform the national hepatitis elimination program, as well as broader eradication plans in resource-limited settings. Using a mixed methods approach that combines a robust methodology (cluster-randomized controlled trial) with a cost-effectiveness analysis and qualitative research, this study seeks to provide a comprehensive view of the impact of such strategies and the feasibility of their implementation and scaling-up.

## Appendix

Multimedia Appendix 1 Study schedule.

Multimedia Appendix 2 Peer review report by France Recherche Nord&Sud Sida-HIV Hépatites (L'ANRS) Maladies infectieuses émergentes (MIE).

## Abbreviations

CAM-C: Community Versus Facility-Based Services to Improve the Screening of Active Hepatitis C Virus Infection in Cambodia
CHW: community health care worker
DAA: direct-acting antiviral
DBS: dried blood spot
HBV: hepatitis B virus
HCV: hepatitis C virus
MSF: Médecins Sans Frontières
OD: operational district
RDT: rapid diagnostic test
WHO: World Health Organization

## Data Availability

The datasets generated during or analyzed during this study are available from the principal investigators upon reasonable request, subject to approval from the National Ethical Cambodian for Health Research.
