# Supplementary material for: Community Versus Facility-Based Services to Improve the Screening of Active Hepatitis C Virus Infection in Cambodia: The ANRS 12384 CAM-C Cluster Randomized Controlled Trial—Protocol for a Mixed Methods Study
Source: JMIR Res Protoc. 2024 Nov 20;13:e63376. doi: 10.2196/63376 (PMC11618004; doi:10.2196/63376)
Supplement: Multimedia Appendix 1 [file resprot_v13i1e63376_app1.docx]

|  | Testing phase | Therapeutic phase for positive HCV RNA | W4 | W8 | W12 | W24 | W28 |
| --- | --- | --- | --- | --- | --- | --- | --- |
| Consent form | X | X |  |  |  |  |  |
| Eligibility criteria | X | x |  |  |  |  |  |
| Consultation and clinical exam |  | x | x | x | x | x | x |
| HIV RDT |  | x |  |  |  |  |  |
| HBs Ag RDT |  | x |  |  |  |  |  |
| HCV Ab RDT | X |  |  |  |  |  |  |
| HCV RNA plasma | x ^a^ |  |  |  |  | x |  |
| HCV RNA DBS | x ^b^ |  |  |  |  |  |  |
| AST / ALT |  | x | x | x | x | x |  |
| Creatinine |  | x | x | x | x |  |  |
| CBC |  | x | x |  |  | x |  |
| Pregnancy test^d^ |  | x | x | x |  |  |  |
| PT |  | x |  |  |  | X^c^ |  |
| Bilirubin |  | x |  |  |  | X^c^ |  |
| Albumin |  | x |  |  |  | X^c^ |  |
| Fasting glycemia |  | x |  |  |  |  |  |
| Score APRI / FIB4 |  | x |  |  |  |  |  |
| Liver ultra-sound |  | x |  |  |  | X^c^ |  |
| Biobank |  | x |  |  | x | x |  |
| Socio-economic evaluation | x |  |  |  |  |  |  |
| DAA delivery |  | x | x | x |  |  |  |
| Adherence assessment |  |  | x | x | x |  |  |

(a) For patients in arm 1

(b) For patients in arm 2

(c) For cirrhotic patients

(d) Pregnancy test will be performed at inclusion, W4 and W8 for all women of childbearing age
