# Supplementary material for: Community Versus Facility-Based Services to Improve the Screening of Active Hepatitis C Virus Infection in Cambodia: The ANRS 12384 CAM-C Cluster Randomized Controlled Trial—Protocol for a Mixed Methods Study
Source: JMIR Res Protoc. 2024 Nov 20;13:e63376. doi: 10.2196/63376 (PMC11618004; doi:10.2196/63376)
Supplement: Multimedia Appendix 2 [file resprot_v13i1e63376_app2.pdf]

ANRS - GRILLE D'EVALUATION Projet – AàP 2018-1

CSS 6 - Recherches dans les pays à ressources limitées

Demandeur	Expert
DUCLOS-VALLEE Jean-Charles / SAPHONN Vonthanak	Rapporteur A

N° de dossier : ECTZ62172

Titre : Approche communautaire versus fondée sur les établissements de santé pour améliorer le dépistage de l'infection active par le VHC au Cambodge: une étude randomisée contrôlée en grappes

---

PARTIE I : Evaluation Scientifique

- **Le projet relève-t-il des missions scientifiques de l'ANRS ?** **oui**
- **Qualité du projet (scientifique et technique)**

Pertinence pour la recherche sur l'infection à VIH, les hépatites virales et/ou co-infections	**Excellent**
Progrès par rapport à l'état actuel des connaissances	**Bon**
Définition des hypothèses et des objectifs	**Bon**
Pertinence de l'approche méthodologique, statistique et/ou technologique	**Bon**
Impact potentiel du projet	**Bon**

- **Faisabilité du projet**

Selon les personnels chercheurs et techniciens du laboratoire	**Bon**
Réalité du partenariat avec les équipes extérieures associées au projet	**Excellent**
Adéquation de l'infrastructure (y compris conditions de sécurité)	**Bon**
Acquis du laboratoire (publications, etc.)	**Bon**

PARTIE II : Adéquation budget / projet

- **Coûts**

Fonctionnement	**Raisonnable**
Equipement (< 16 000 euros HT)	**NonApplicable**
Personnel	**Raisonnable**
Participation demandée à l'ANRS	**Raisonnable**
Coût total estimé du projet	**Raisonnable**

PARTIE III : Dimension éthique

Les problèmes éthiques ont-ils été pris en considération (homme, animal) ?	**Oui**
Si le projet relève de la règlementation applicable à la recherche sur la personne, les demandes nécessaires ont-elles été prises en considération?	**Oui**

PARTIE IV : Respect de la charte d'éthique de la recherche dans les pays en développement

	**Ce sujet est-il abordé dans le projet ?**	**Si oui, de façon satisfaisante ?**
L'impact potentiel de la recherche pour la collectivité en terme de santé publique est-il envisagé ?	**Oui**	**Bon**
Le rapport bénéfice-risque pour la personne participante est-il évalué ?	**Oui**	**Bon**
Des moyens pour assurer la confidentialité sont-ils pris ?<br>(confidentialité liée à la séropositivité, aux données personnelles, …)	**Oui**	**Bon**
Un médecin référent sera-t-il désigné pour chaque participant ?	**Oui**	**Bon**
La constitution d'un comité indépendant est-elle prévue ?	**Oui**	**Bon**
Des moyens pour éviter les conséquences discriminatoires ou stigmatisantes de la recherche sont-ils pris ?	**Non**	**NonApplicable**
Un counselling pré et post test de dépistage est-il prévu ?	**Non**	**NonApplicable**
La prise en charge médicale pendant la recherche est-elle assurée ?<br>(par le projet, par le système de santé du pays, etc…)	**Oui**	**Bon**
Les conditions de prise en charge post-recherche sont-elles définies ?	**Oui**	**Bon**
Des moyens pour communiquer les résultats de la recherche aux participants sont-ils définis ?	**Oui**	**Bon**
Les bénéfices de la recherche seront-ils rendus accessibles à la personne participante ?	**Oui**	**Moyen**
Des représentants qualifiés de la communauté ou des associations de personnes vivant avec le VIH ou une hépatite virale sont-ils impliqués dans la mise en place et le déroulement de ce projet de recherche ?	**Non**	**NonApplicable**
Si une notice d'information et/ou un formulaire de consentement sont fournis, leurs contenus vous paraissent-ils adaptés ?	**Oui**	**Bon**

RAPPORT

Il s'agit d'une soumission d'un projet de recherche porté par Jean Charles Duclos Vallee de l'Inserm U785 (Hôpital Paul Brousse) et Vonthanak Saphonn de l'université des sciences de la santé de Phnom Penh, Cambodge visant à comparer deux stratégies de dépistage combiné (anticorps + ARN VHC) de l'infection par le VHC au Cambodge : une stratégie avec une approche communautaire versus une approche fondée sur les établissements de santé.
La prévalence par HCV dans la population générale est comprise entre 3 et 5%. La population générale âgée de plus de 40 ans est particulièrement touchée en raison d'une transmission nosocomiale avant les années 2000. De plus, cette population est plus à risque de fibrose sévère et cancer du foie. C'est cette tranche de la population qui est visée par cette étude.
Méthode : Il s'agit d'une étude randomisée contrôlée en grappes. Le 1er bras correspond à l'approche utilisant les établissements de santé ; des tests de diagnostiques rapides (TDR) Ac VHC seront à disposition des centres de santé et la communauté en sera informée. Si le TDR est positif, un prélèvement de sang sera effectué et envoyé à l'hôpital du district pour extraction de l'ARN par technique Xpert sur plasma et les résultats renvoyés aux centres de santé.
Le 2ème bras correspond à l'approche communautaire : une équipe mobile parcourra les villages pour proposer le test rapide de diagnostique directement, et si le résultat est positif, un DBS (goutte de sang sur papier buvard) sera envoyé à l'hôpital national à Phnom Penh pour l'extraction et amplification de l'ARN, car accès limité à des laboratoires permettant le test virologique sur plasma sanguin (problème de stockage, transport..). Le résultat sera renvoyé à l'équipe mobile.
Tous les patients ARN VHC +, s'ils ne présentent pas de cirrhose symptomatique, quel que soit le bras dans lequel leur village a été randomisé, seront référés à l'hôpital du district et un traitement par antiviraux à action directe AAD sera proposé immédiatement et systématiquement, avec une évaluation hépatique allégée (combinaison sofosbuvir/daclatasvir pour 12 semaines). Ceci afin d'accélérer la mise sous traitement. La prise en charge des patients avec cirrhose symptomatique et les patients non répondeurs sera faite à l'hôpital national pour une évaluation hépatique plus approfondie.
L'objectif principal de cette étude est de comparer l'efficacité de ces 2 stratégies de dépistage combiné (Ac+ARN VHC) sur la couverture de dépistage des cas chez les plus de 40 ans.
Les objectifs secondaires sont :

- comparer les taux de détection avec infection active de cas entre les 2 stratégies (ac+ et ARN VHC+) chez les plus de 40 ans
- comparer la cascade de soins entre les 2 bras (au moins 1 consultation à l'hôpital de district) parmi les ARN VHC +
- comparer l'accès au traitement parmi les ARN VHC +
- comparer l'efficacité du traitement entre les 2 bras (maintien de la réponse virologique 12 semaines après l'arrêt du traitement)
- évaluer le cout-efficacité des deux stratégies (stratégie mobile de test HCV, et l'introduction de l'Xpert dans les hôpitaux de district)
- comparer la morbidité (liée au foie) et la mortalité entre les 2 bras à la fin de l'étude (28 semaines)

Nombre de sujets : l'hypothèse est basée sur une augmentation de 20 points de la couverture de dépistage combinée (60% vs 80%). Afin de montrer cette différence entre les 2 stratégies, avec puissance de 90%, un risque alpha 5%, 240 personnes HCV Ac+ devront être inclues, correspondant à 60 grappes (30 par bras) constituées de 50 foyers de 4 personnes (200 personnes) d'un même village. Au total, 3500 individus âgés de plus de 40 ans seront testés par TDR.

Points forts:
Originalité de l'étude (approche communautaire et mise sous traitement immédiate et quasi systématique) incluant une étude coût-efficacité et aussi une évaluation socio-economique des patients

pour la pérénité de cette stratégie (étude d’une possibilité de microcrédit), dans l'optique sous jacant de révision des guidelines nationaux.
Projet très bien écrit et très clair

Points faibles:

- Partie statistique : uniquement le calcul de la taille d'échantillon sinon elle est inexistante. Pas de détails sur les analyses prévues, ni les conventions à prendre sur les population d'analyse, si les patients ont déjà été diagnostiqués ou sous traitement par exemple?
- L’estimation des prévalences, sérologie et virologique non abordée
- Les tests virologiques sont differents pour les 2 bras : comparaisons des tests ou des approches ?
- Volet de modélisation de l'épidemie HCV dans le pays non détaillée
- Estimation du nombre de patients qui vont être traités ? Dans le budget, achat de traitement pour environ 150 patients ?
- Budget équipe B : des lignes pour lesquels les postes ne sont pas décrits ; à détailler donc, au vu du nombre de participants dans l'étude (enqueteur, saisie, ARC… ? personnel hospitalier ? nurse ?) pas seulement depistage mais pour le suivi aussi.

CONCLUSION

Projet bien écrit sur un sujet pertinent et original. Des parties à détailler notamment sur les aspects statistiques.

NOTATION GENERALE

BAvisFavorable
